# Experimental and Numerical Study on the PG-7VM Warhead Performance against High-Hardness Armor Steel

**DOI:** 10.3390/ma14113020

**Published:** 2021-06-02

**Authors:** Paweł Żochowski, Radosław Warchoł, Maciej Miszczak, Marcin Nita, Zygmunt Pankowski, Marcin Bajkowski

**Affiliations:** 1 Military Institute of Armament Technology, 7 Wyszynskiego Street, 05-220 Zielonka, Poland; warcholr@witu.mil.pl (R.W.); miszczakm@witu.mil.pl (M.M.); nitam@witu.mil.pl (M.N.); pankowskiz@witu.mil.pl (Z.P.); 2 Faculty of Production Engineering, Institute of Mechanics and Printing, Warsaw University of Technology, Narbutta 85 Street, 02-524 Warsaw, Poland

**Keywords:** shaped charge, shaped charge jet, high-explosive anti-tank warhead, PG-7 grenade, high-hardness armored steel, numerical simulation, penetration

## Abstract

Analyses presented in the article were carried out in order to characterize the main parameters of the shaped charge jet formed due to detonation of the PG-7VM warhead. As opposed to the previously published studies in which rolled homogeneous armored steel was mainly used as a target, in the current work the warhead penetration capability was determined against more contemporary high-hardness (500 HB) ARMSTAL 30PM steel armor with precisely determined mechanical properties. The research included experimental depth of penetration tests and their numerical reproduction in the LS-Dyna software. Special attention was paid to factors that could perturbate the shaped charge jet formation process and under- or overestimate its penetration capability. For this reason, warheads were X-ray inspected for structural discrepancies (voids or air inclusions in explosive, misalignment between the body, explosive, and liner, or lack of contact between the explosive and the liner) and properties of materials (explosive, targets, and most important warhead components) were analyzed before the experiments. The numerical model of the warhead was defined more accurately than in previously published studies, since it was based on the real grenade dimensions and its technical documentation. Thanks to this, the depth of penetration of the target made of ARMSTAL 30PM armored steel plates by the shaped charge jet formed from the PG-7VM warhead obtained by numerical simulation was consistent with the experimental results and equaled 278 mm and 280 mm, respectively. The difference between the experimental and numerical value was smaller than 1%, which confirms that the developed methodology of modeling allows users to properly reproduce the PG-7VM shaped charge jet formation and target penetration processes. A verified numerical model of the shaped charge jet penetration into a steel target was used to determine depth of penetration in function of stand-off distance for the PG-7VM warhead. A maximum depth of penetration of about 317 mm was obtained for the stand-off distance of 360 mm, which may indicate the potential direction of modernization of warheads.

## 1. Introduction

High-explosive anti-tank (HEAT) warheads [1,2,3,4], besides the armor-piercing fin stabilized discarding sabots projectiles (APFSDS) [4,5,6], are one of the most effective types of anti-tank ammunition currently used by armed forces worldwide. The high effectiveness of HEAT warheads results from the presence of shaped charges (SCs) in their design. The fundamentals of SCs have already been well understood and described [2,3]. An SC basically employs a highly explosive device with a lined cavity. The liner is typically a hollow metal cone but may assume almost any geometrical shape [7]. The cavity focuses the energy of the detonation products on a small area. The collapsing liner forms a high-velocity jet that has a great penetration capability. Therefore, SCs are also used in various branches of industry where deep perforation or precise cutting is required, e.g., demolition works [8,9], mining [10,11], etc.

Recently developed and technologically advanced military HEAT warheads have sophisticated designs, allowing them to obtain a great penetration capability of armor. Depending on the type (single, tandem, or even triple SC arrangements) the most powerful HEAT warheads may penetrate up to 1500 mm of rolled homogeneous armor (RHA) [4]. However, together with the technological advancement, the accessibility of such sophisticated SC warheads significantly decreases, mainly due to their high costs of production and difficulties in their manual usage, which requires a lot of knowledge and training. In consequence, modern weapons using advanced HEAT warheads are less common and paradoxically constitute a lower threat than their elder, simpler, and less effective equivalents. Features such as low cost, functioning reliability, action efficiency, availability, and easy usage that does not require a lot of training make the old (invented more than 50 years ago) and simple RPG-7 the most widely used shoulder-fired antitank weapon in the world today [12,13]. Various types of RPG’s, but mainly RPG-7, are still being produced in a few countries (e.g., in Russia, China, Egypt, Pakistan) [12] and are still in active use by at least 90 national armed forces and various terrorists organizations and guerrilla groups around the world. According to statistics, the injuries caused by RPG-7 attacks on vehicles were the second (after IED—improvised explosive device—detonations) most frequent reason of deaths of soldiers in Iraq and Afghanistan [14]. The US Army forces have lost more vehicles and personnel to the PG-7 hits than to more advanced modern guided missiles [12]. Neglecting the reliability of this data, there is no doubt that the PG-7 HEAT warheads were the most frequently used in recent military conflicts and they still constitute a serious threat.

Effectiveness of HEAT warheads is determined mainly during experimental depth of penetration (DoP) tests. However, currently used measurement techniques, which are based mainly on flash radiography and X-ray pictures of the SCJ at predetermined times, are unable to provide all the necessary data about the entire phenomenon. Although, X-ray pictures may be subsequently used as input data for the analytical models, allowing calculation of the basic SCJ parameters, such a combined experimental–analytical approach is effective only in analysis of the simplest penetration scenarios. For this reason, numerical simulations are often used as a support to experimental analyses. Numerical methods allow researchers to collect more information about the entire process of SCJ formation and penetration, e.g., pressure, density, or velocity distribution at different times. Nowadays, a few different modeling approaches are used in analysis of SCJs. In the work [15], a Lagrangian solver was used to reproduce an entire process of the SCJ–target interaction: from the SCJ formation to the penetration of a plate. Two-dimensional Euler code with multi-material interface treatment was used to investigate the effect of the liner material of the SC on SCJ formation and its penetration capability [16,17]. The penetration of targets by SCJ with the assumption that the SCJ is a high-speed rod with a large velocity gradient was analyzed in the work [18]. The three-dimensional multi-material Euler solver in AUTODYN^®^ software was used to investigate the influence of SC asymmetries on the SCJ characteristics in the work [19]. Two-dimensional smoothed particle hydrodynamics (SPH) method was used in the work [20] to simulate the detonation process of a SC. However, the authors could not avoid the specific SPH problem with the interfaces of different materials’ treatment. On the basis of the literature review, it can be concluded that a combined experimental and numerical approach can be very effective in testing and designing of HEAT warheads.

Although, multiple studies on the effectiveness of various HEAT warheads with different numbers of SCs [21,22], liner shapes [23,24,25], liner materials [26,27,28], and types of explosives used [29] were presented in the literature, paradoxically there is a lack of accurate and reliable research related to the performance of the most common PG-7VM warhead. In the available studies related to PG-7VM efficiency, the geometry of the warhead is often modeled in an inappropriate or too simplified way and the results are usually limited to the value DoP in RHA steel. Moreover, the DoP values that can be found in various publications significantly differ and depending on the source are: 300 mm [30], 330 mm [31], up to 400 mm of RHA (probably for the Bulgarian version of the grenade labelled PG-7VN, which has very similar design and may be confused with PG-7VM) [4,32]. One of the reasons for such a big spread of PG-7VM penetration capability may be the fact that properties of RHA steel (which is commonly used as a reference target) are strongly dependent on the thickness of the plates. The hardness of the RHA plate with the thickness of 300 mm is two times lower than the RHA plate of 20 mm thickness [33,34]. The reliability of the literature data is questionable also due to the fact that the referred values of penetration capability are usually not confirmed by any pictures of craters in the penetrated target.

An additional issue is the relevance of RHA as a reference material simulating passive armors of modern heavy-armored vehicles. RHA has been used as a reference target for over 50 years and its main advantage is that effectiveness of various warheads can be easily compared on the basis of the values of DoP in RHA. However, during those 50 years, technology of production of armored steels has significantly improved and RHA steel was almost completely replaced with new, more durable, and harder steel grades. Strength and hardness of currently offered armored steel is almost twice that of RHA (hardness: RHA-250-370 HB, Armox 500T-500 HB, Armox 600T-600 HB, Armox Advance 640-720 HB) [35]. Taking into consideration the information available in the literature that the SCJ penetration in RHA plate is 10–15% [36] or even 20% [37] less than in mild steel, it can be assumed that the penetration in recent high-hardness armored steel will be significantly lower than in RHA. However, in the literature, there is a lack of research related to the penetration capability of the PG-7VM warhead against more contemporary high-hardness (≥500 HB) armored steels. In the majority of previously published studies the data obtained from the experiments were limited to the value of depth of penetration of RHA by the PG-7VM warhead. The process of shaped charge jet formation, material properties of individual components, as well as the precision of the warhead assembly were not sufficiently examined in regards to what caused the large spread of the obtained results and decreased their reliability [30,31,32].

In view of the foregoing considerations, the main aim of the work presented in the article was evaluation of the actual PG-7VM warhead performance against the target made of high-hardness (500 HB) ARMSTAL 30PM armored steel [38] with precisely defined mechanical properties. Firstly, experimental DoP tests were carried out to determine the warhead penetration capability and to collect data required for validation of the numerical model of the phenomenon. Before the experiments, the presence of structural discrepancies in the warheads were X-ray inspected and material properties of explosive, target, and most important warhead components were analyzed during material characterization tests to exclude all the factors that could negatively affect (under- or overestimate) warhead penetration capability. Simulations were carried out in order to collect more detailed information related to SCJ parameters during the SCJ formation and target penetration process and to determine the basic characteristics of the warhead: DoP in function of stand-off distance (SoD), defined as a distance between the base of the liner and the target.

Effective and reliable methodology of numerical modeling of shaped charge jet penetration into high-hardness armored steel targets could be used in the future in all kind of optimization studies of both warheads and armor designs.

## 2. Materials and Experimental Methods

### 2.1. Experimental Methodology

**Figure 1:**
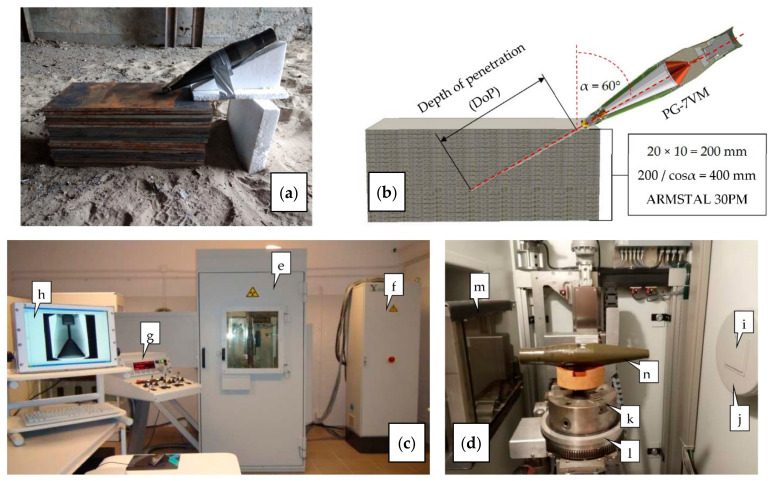
Experimental test setup: (**a**) real setup of DoP tests; (**b**) schematic setup of DoP tests with some geometrical and material data; (**c**) X-ray Diagnostic System MU17F 225-9 (by YXLON International GmbH, Hamburg, Germany) used for the inspection of PG-7VM warheads; (**d**) interior of the radiation cabin; (**e**) radiation cabin with the HDR detector and the X-ray tube inside; (**f**) power supply device; (**g**) control panel with a joystick; (**h**) monitor with a real-time view of the warhead; (**i**) adjustable shutter; (**j**) X-ray lamp; (**k**) three-jaw chuck; (**l**) turning table; (**m**) flat panel detector; (**n**) inspected PG-7VM warhead.

Experimental DoP tests were carried out to determine the warhead penetration capability and to collect data required for further validation of the numerical model of the phenomenon. The experiments were performed at the Military Institute of Armament Technology (MIAT) proving ground in Poland. During the tests, the PG-7VM warheads were placed at an angle of α = 60° in relation to the vector normal to the surface of the target (Figure 1) in order to limit the number of plates required to provide the desired thickness of the target (ensuring stopping of the SCJ). The target consisted of stack of twenty 10 mm thick ARMSTAL 30PM high-hardness (500 HB) armored steel plates [38]. The warheads were statically initiated. Three detonations were performed. The penetration capability of the PG-VM warhead was determined on the basis of the measured depths of penetration craters in the target. Dimensions of these craters constituted reference values used for further verification of the developed numerical model. The experimental test setup is shown in Figure 1.

In order to increase the reliability of the results before the experiments, the PG-7VM warheads selected for the tests were X-ray inspected for their structural and geometrical discrepancies (e.g., voids or air inclusions in explosive; misalignment between the body, explosive, and liner; lack of proper contact between the explosive and the liner) to exclude some factors that could perturbate the jet formation and penetration process. The X-ray real-time radioscopy (RTR) diagnostic system (XDS) MU17F 225-9 (by YXLON International GmbH, Hamburg, Germany) was used (Figure 1c,d).

**Figure 2:**
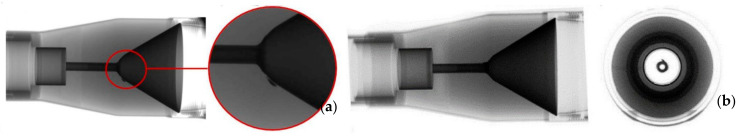
Examples of RTR X-ray images of a PG-7VM warhead showing its axial and radial cross-section: (**a**) foreign body in explosive; (**b**) warhead without discrepancies.

The X-ray inspection is particularly important because the effectiveness of the PG-7VM warhead is highly dependent on the axial alignment precision of their elements during the manufacturing and assembling process. The most effective (in terms of penetration capability of armor) are warheads in which the dimensional deviations from axial symmetry of liner and explosive do not exceed 0.03 mm. The penetration capability of the warhead significantly decreases with the increase of inaccuracy of production or assembling. Depending on the deformation rate of the liner and the high explosive charge, the process of SCJ formation will be disturbed or will not occur at all. Effects of the warhead’s influence on the target are then comparable to the simple detonation of explosive charge contained in the warhead body. Only the warheads with no discrepancies (e.g., foreign bodies in explosives Figure 2a) were selected to test.

### 2.2. Materials and Components

#### 2.2.1. PG-7VM Warhead

**Figure 3:**
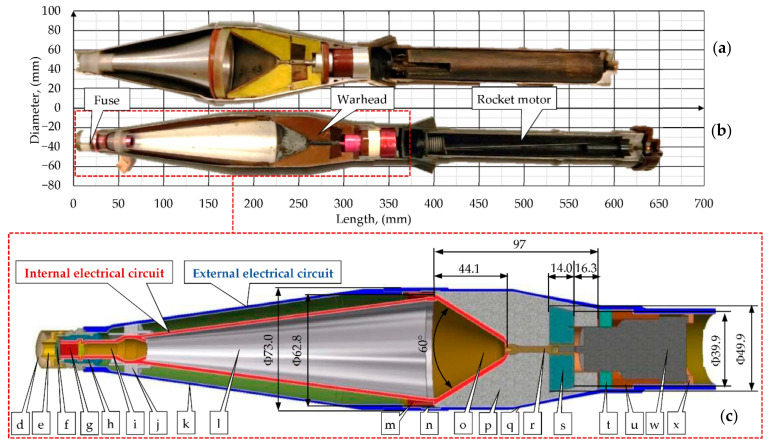
Comparison of the PG-7 warheads: (**a**) standard PG-7V and (**b**) modernized PG-7VM. (**c**) The cross-section and dimensions of the main components of the PG-7VM warhead: (**d**) fuse cover; (**e**) nut; (**f**) joint; (**g**) piezoelectric element; (**h**) isolator; (**i**) fuse body; (**j**) isolator sleeve; (**k**) ballistic cap; (**l**) conductive cone; (**m**) lock ring; (**n**) isolator ring; (**o**) liner; (**p**) explosive; (**q**) warhead body; (**r**) conductor; (**s**) cover; (**t**) isolator; (**u**) sleeve; (**w**) bottom part of the fuse; (**x**) lock ring.

The PG-7VM grenade (Figure 3) was analyzed in the study, which is a modernized version of the standard PG-7V type grenade (Figure 3a) with increased penetration capability, lower sensitiveness to side winds, and increased muzzle velocity (“M” in Russian means modernizirovanyy—modernized). The PG-7VM grenade introduced into the Soviet Army in the 1970s has a smaller diameter (70 mm) and is longer (by 52 mm) than the standard one (PG-7V). Due to the fact that in the available studies related to PG-7VM efficiency, the geometry of the warhead is often modeled in an inappropriate or too simplified way, the dimensions of the main components of the warhead are presented in Figure 3.

The PG-7VM grenade consists of four main components: fuse, warhead, rocket motor, and flight stabilizer. The grenade flies with a velocity of 200–250 m/s. During impact into the target, the piezoelectric element, which is located in the head part of the fuse is compressed and generates an electrical impulse. Inside the grenade, there are two electrical circuits: external and internal (Figure 3). The electrical impulse is transmitted by the internal circuit to the bottom part of the fuse and the explosive detonates. The generated detonation wave acts on the liner material and causes the formation of a high speed SCJ (2–10 km/s).

#### 2.2.2. ARMSTAL 30PM Armored Steel

In order to determine the PG-7VM warhead’s penetration capability against modern high-hardness armored steel, ARMSTAL 30PM steel plates were used as a target during the experiments. The material is manufactured by “Huta Stali Jakościowych” steel mill in Poland [38]. ARMSTAL 30PM is a high-hardness (500 HB) armored steel used mostly in ballistic armor applications. The chemical composition of the steel is shown in Table 1 [35].

The chemical composition combined with properly adjusted heat treatment allow manufacturers to obtain high strength and ductility of the material. According to the manufacturer datasheet, the mechanical properties of the ARMSTAL 30PM steel are similar to other high-hardness armored steel grades of 500 HB class (Table 2).

**Figure 4:**
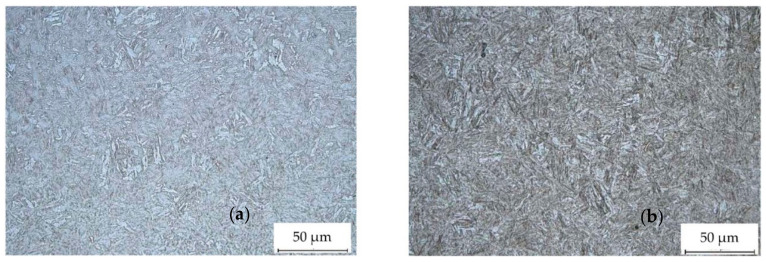
Microstructure of the 30PM steel: (**a**) frontal surface; (**b**) cross-section.

Microscopic inspection showed that microstructure of ARMSTAL 30PM is highly uniform throughout the thickness of the plate (Figure 4).

Material characterization tests were carried out on the samples of ARMSTAL 30PM in order to determine the response of the material to specific loads and to collect data (force–displacement curves) required for subsequent definition and validation of the numerical model of the material. The following tests were carried out:

- Hardness measurement;
- Quasi-static tension and compression tests;
- Impact strength tests.

**Figure 5:**
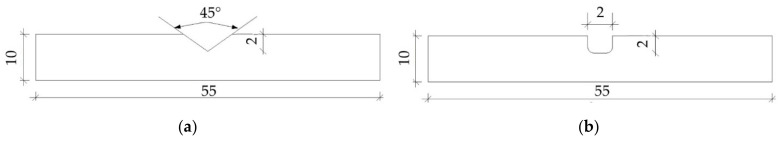
Geometry of the samples during impact strength tests (**a**) V-notch shape; (**b**) U-notch shape.

Hardness measurements were carried out with the use of a Brinell machine, both parallel and perpendicular to the rolling direction (EN ISO 6506-1). Before the measurements, the surfaces of the plate were polished. Impact strength of ARMSTAL 30PM was tested according to well-known Charpy method (EN 10 045-1). Tests were carried out for the samples with U- and V- shape notches at temperatures of +20 °C and −40 °C, respectively. The geometry of the samples is presented in Figure 5. The results of ARMSTAL 30PM hardness measurements and impact strength tests are shown in Table 3.

Mechanical properties of the ARMSTAL 30PM were analyzed on a Zwick Z100 universal testing machine (by ZwickRoell GmbH & Co. KG, Ulm, Germany) with servo-hydraulic control. Specimens for the tests were prepared from 4, 6, 8, and 10 mm thick plates using a wire electrical discharge machine. Geometry of the samples prepared for the tests were shown in Table 4.

Force was applied to the specimen holders with end grippers and their displacements were recorded. Engineering stresses and strains values were calculated on the basis of the force–displacement curves. After necking or barreling of the sample during tension and compression tests, respectively, diversified, three-dimensional stress states in the samples were generated. The barreling effect during compression tests is caused by friction between the sample and the surface of the specimen holders. Various stress states can be distinguished in individual areas of the sample:

1. Triaxial, close to hydrostatic compression in the sample/holder interface zones;
2. Triaxial non-homogeneous compression in the middle layers of the sample;
3. Mixed compressive tensile state in the middle subsurface layers of the sample with a tensile stress in the circumferential direction.

Due to barreling and necking effects, determination of the true stresses and strains in the sample requires continuous measurement of the actual cross-section of the sample, e.g., by means of digital image correlation equipment. In the study, the compression and tension processes were recorded with a camera and the values of actual cross-sections of the samples were calculated from the recordings on the basis of the extremal lateral dimension of the tested samples, located usually in the middle of their height, where the maximum deformation occurs. Assuming negligible changes in the volume of the samples, the true stresses and strains were calculated from the following equations:

(1) $$\sigma_{\mathrm{true}}=\frac{P}{A_{i}}$$

(2) $$\varepsilon_{\mathrm{true}}=2\ln\frac{A_{i}}{A_{0}}$$

where: σ_true_—true stress, ε_true_—true strain, P—force, A_i_—area of actual cross-section of the sample, A_0_–area of initial cross-section of the sample.

**Figure 6:**
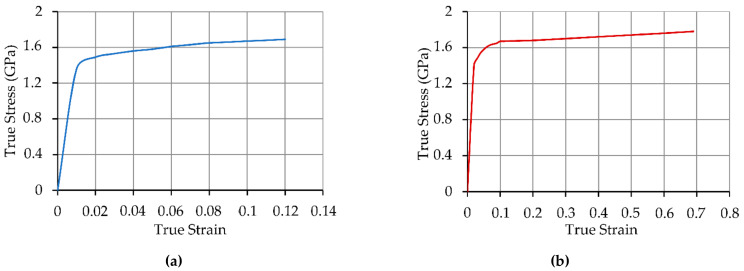
True stress–strain curves of the ARMSTAL 30PM calculated on the basis of mechanical test results (strain rate 10^−3^ s^−1^): (**a**) tension; (**b**) compression.

The results of quasi-static compression tests of the ARMSTAL 30PM steel are shown in Table 5 and Figure 6.

Mechanical properties of ARMSTAL 30PM obtained during the tests on the samples taken parallel and perpendicular to the rolling direction were similar to what is known about the high uniformity and low anisotropy of the material. The differences in the results obtained for the samples prepared from the plates with different thickness were also very small. The yield strength and ultimate tensile strength of the material exceeded the minimal values that were provided in the manufacturer datasheet (Table 2). On the other hand those values were a little bit lower than in the works [40,41,42], in which authors investigated the behavior of ARMSTAL 30PM using a combination of quasi-static and dynamic tests for a wide strain rate range. Determined true stress–strain curves of the ARMSTAL 30PM were used to define the numerical model of the material.

#### 2.2.3. A-IX-1 Explosive Composition

**Figure 7:**
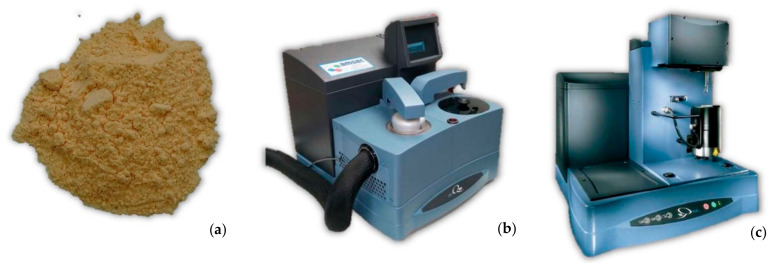
A-IX-1 explosive composition extracted from the PG-7VM warhead (**a**) to test on a differential scanning calorimeter DSC Q100 (**b**) and thermogravimetric analyzer TG Q50 (**c**).

PG-7V and PG-7VM warheads are filled with an A-IX-1 explosive composition (phlegmatized RDX), which consists of 94–96% RDX (hexogen) and 4–6% wax. More modern warheads are filled with OKFOL explosive composition, which consist of 96% HMX and 4% wax [12]. Explosives that are subjected to extended storage and exposure to weather conditions (temperature amplitude, humidity, etc.) may lose their properties. Therefore, in order to exclude the highest possible number of factors that could negatively affect (under- or overestimate) warhead penetration capability, the main parameters of A-IX-1 explosive composition were analyzed. The explosive was extracted from the warhead with a hydraulic press and non-sparking tools (Figure 7a). In order to increase the reliability of the results, samples to test were extracted from three warheads selected from different production batches: X–90–406, Y–85–406, Z–78–406.

Phlegmatized hexogen was subjected to the following tests:

- Differential scanning calorimetry (DSC) analysis performed with the use of DSC Q100 apparatus of TA Instruments (Eschborn, Germany);
- Thermogravimetry (TGA) analysis, performed with the use of the TG Q50 apparatus of TA Instruments (Eschborn, Germany);
- Pycnometric density analysis, performed with the use of the Ultrapyc 1200e by Quanta Chrome Instruments (Graz, Austria);
- Moisture analysis, performed with the use of glass weighing vessels and chamber thermostats with a water jacket;
- Stability analysis carried out with the use of glass weighing vessels and chamber thermostats with a water jacket.

**Figure 8:**
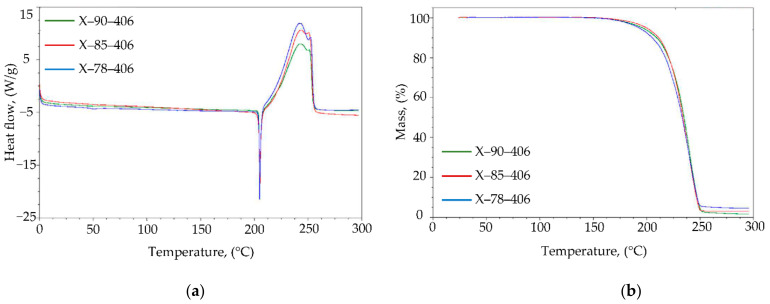
DSC (**a**) and TGA (**b**) curves obtained for samples of A-IX-1 material extracted from PG-7 warheads.

The results of DSC and TGA analyses for the tested samples of phlegmatized hexogen (A-IX-1) are shown in Figure 8 and in Table 6. Characteristic thermal parameters are shown in the Table 6, such as: T_onset_—temperature at transformation start; T_max_—temperature at maximum peak, in which the process takes place at the highest rate; T_end_—temperature at end of transformation; Δm—mass loss of the tested sample in percents; Q—heat of transformation (J/g). The tests were performed in non-hermetic aluminum vessels (with a lid), in a nitrogen atmosphere, with a heating rate of 10 °C/min.

On the basis of the obtained results, it can be concluded that the temperature of the beginning of the melting process of the A-IX-1 explosive composition is within the range of 204.0 to 204.3 °C. The T_onset_ temperature of the thermal decomposition process determined with extrapolation were 217.1–219.5 °C and 217.2–221.0 °C for DSC and TGA analyses respectively. The largest differences were observed in the values of the thermal decomposition of hexogen contained in the tested composition (1986–2507 J/g). The decomposition process is a highly exothermic transformation, during which, in the case of non-hermetic vessels, a partial loss of gaseous products may occur (through the upper part of the measuring vessel). The mass loss of the tested explosive samples was in the range from 95.9% to 99.0%. The differences in the recorded mass may result from the amount of wax in a given production batch of A-IX-1 composition. On the basis of the authors experience, it may be assumed that the content of stearin or ceresin in the composition A-IX-1 may vary within ±2%, which was confirmed by the results of TGA analyses.

The results of the density determination with the pycnometric method, as well as humidity and stability by the weight method, are presented in Table 7. The mass of the explosive samples subjected to the pycnometric and weight tests were 1.3–1.4 g and about 5 g, respectively.

The measured density of the A-IX-1 composition extracted from PG-7VM warhead ranged from 1.70 to 1.73 g/cm3. The batch-to-batch differences may be caused by the wax content. The higher the wax content, the lower the density value. The results of the humidity and stability tests for all tested samples were below 0.05% and 0.1%, respectively. However, it should be noted that the density of the charge is always lower than the density of the extracted composition powder due to the limitations of the pressing process. Theoretical density of composition containing 96% of RDX and 4% of wax may reach up to 1.78 g/cm^3^. Actual density of the charge after the pressing process, which is indicated in the PG-7VM warhead technical documentation, is 1.63 g/cm^3^. Since the results of performed physicochemical tests were similar (even though samples of explosive compositions were extracted from warheads from different production batches and year of manufacture), it can be assumed that that the explosive compositions extracted from the PG-7VM warheads have not undergone excessive aging due to the long storage and should have properties similar to those reported in the manufacturer datasheet. Therefore, the density of the charge was assumed as 1.63 g/cm^3^, which gives the detonation velocity of about 8200 m/s according to the relationship between the detonation velocity and density of the phlegmatized RDX, available in the literature and presented in Table 8 [43,44]. These values determined on the basis of the physicochemical tests results and literature data were used to define the parameters of the material model of the explosive composition. It was assumed that the density of the compressed charges in the PG-7VM warhead was equal to this reported in the warhead technical documentation.

## 3. Methodology of Numerical Simulations

Numerical simulations may constitute an effective tool supporting experimental studies of high-speed phenomena that are inconvenient to analyze with the use of traditional measuring techniques. However, the reliability of the results of simulations is strongly dependent on the accuracy of the model. In the literature, there is a lack of reliable numerical studies related to PG-7VM efficiency. In those that are available, the geometry of the warhead is often modeled in an inappropriate or too simplified way and the results are usually limited to the value of DoP in RHA steel. One of the main objectives of this study was to develop an effective and accurate methodology of modeling shaped charge jet penetration into high-hardness armored steel targets. Numerical simulations were carried out in order to better understand the mechanisms of the phenomenon and to collect data related to SCJ parameters during the SCJ formation and target penetration process.

### 3.1. Discretization, Contacts, Initial, and Boundary Conditions

Numerical simulations of the PG-7VM SCJ formation and target penetration processes were carried out in the LS-Dyna software [45]. A three-dimensional (3D) finite element model was developed with a coupled Lagrangian and multi-material arbitrary Lagrangian Eulerian (MMALE) element formulation. In simulations, the warhead axis was perpendicular to the target surface, unlike in experiments. Thanks to this, two symmetry planes could be used in the model and only a quarter of the warhead could be modeled, which significantly reduced calculation time. However, in the simulations, the thickness of plates was increased to 20 mm in order to provide the same number of plate–plate interfaces as in experiments (10 mm thick plate at an angle of 60° gives the actual penetration track of 20 mm) The MMALE element formulation allows simulating high strains of materials without the problems related to the mesh distortions. Therefore, the air domain, the explosive, and the liner were described by MMALE element formulation, while the other components (warhead parts and steel target) were modeled by deformable solid elements. Parameters of the MMALE method that were used in the simulations are listed in Table 9. Detailed descriptions of the parameters can be found in the LS-Dyna software manual [45].

**Figure 9:**
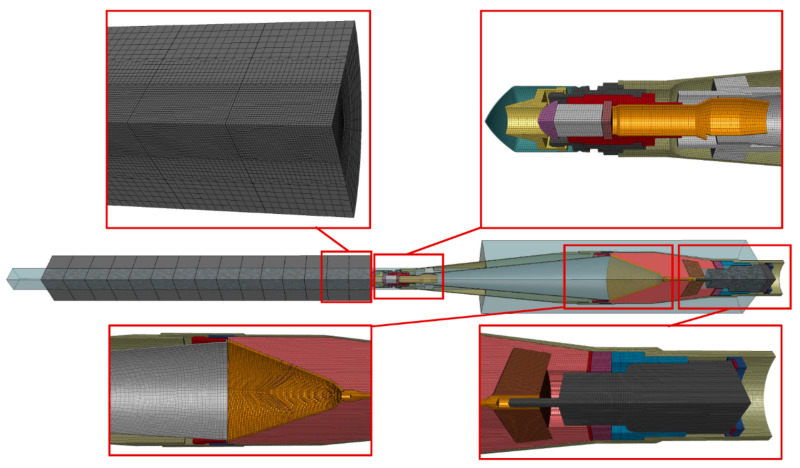
Discretization of the simulation components.

Discretization of the simulation components is shown in Figure 9. It was performed using ALTAIR HyperMesh software. All the components of simulations were meshed by 8-node solid elements with one integration point [45]. Numerical model of the PG-7VM warhead consisted of 23 parts with the appropriate materials assigned. The mesh of the warhead structural components was modeled inside a regular non-deformable finite element mesh for the cylindrical air domain. The elements of the domain had the size of about Δx = Δy = Δz≈0.5 mm. The radius of the domain (50 mm) was larger than the warhead outer radius (D = 30.25 mm), allowing detonation products to expand and to transfer a part of their energy to the warhead casing. Specific elements of the domain were subsequently filled (by replacing the air material) with the liner and explosive materials to reproduce their shapes.

On the external segments of the model, the boundary condition was applied to prevent artificial stress wave reflections generated at the model boundaries from reentering the model and contaminating the results. In the discretization of the target in order to limit the number of elements, the mesh of the individual armor plates was refined in the SCJ impact point zone. The distance between the neighboring nodes ranged from approximately Δx = Δy = Δz = 0.5 mm in the SCJ impact zone to Δx = Δy = Δz = 2 mm in non-deformed armor areas. The elements of the mesh of other components of the warhead had the size of Δx = Δy = Δz = 0.5 mm.

The model of the whole phenomenon is shown in Figure 9. Finally, all the components of the simulation were discretized with 1,602,115 elements (1,217,665 elements for the warhead/domain system and 384,450 elements for the target).

Several contact models, based mainly on the penalty function method [45] and considering the friction between individual components, were used in the analyses. Interactions between the structural components of the simulations (modeled with Lagrange formulation) were conducted by general contact algorithm. In case of components in which damage and erosion was predicted, the eroding contact model was used. Threaded joints were modeled in a simplified manner, through the tiebreak contacts with appropriate values of normal and shear forces that breakaway the joint. In turn the interactions between the MMALE and Lagrange components were conducted by the fluid–structure interaction (FSI) algorithms

### 3.2. Constitutive Equations

Four general types of materials were modeled in the work:

- Metals—steel (target, warhead components), copper (liner, conductor), and aluminum alloys (body, ballistic cap, conductive cone);
- Plastics—wave shaper, separators, etc.;
- Explosive;
- Air filling computational domain.

Since commonly used and well-known constitutive relations available in Ls-Dyna software [46] were used in the simulations, they have not been explained in the article. Details and theory of the constitutive equations and material models used can be found in the literature [46,47,48,49].

The popular material model developed by Johnson and Cook (J-C) [49,50,51,52], together with the Gruneisen equation of state (EOS) [46], were used to describe the behavior of the metallic components of the phenomenon (copper liner, warhead body, and ballistic cap made of aluminum alloys, target made of ARMSTAL 30PM steel). The values of parameters in the equations of metallic components of the PG-7VM warhead that had the biggest influence on the simulation process are presented in Table 10.

Values presented in Table 10 were adopted on the basis of own material characterization tests, as well as data available in the literature and ANSYS Autodyn material library. Parameters for the copper liner were taken as for Cu-OFHC material in ANSYS Autodyn material library [48]. Aluminum alloys were modelled on the basis of both ANSYS Autodyn material library (AL2024T351 material) and the literature data [53,54], in which authors studied the behavior of the EN AW- 7012 aluminum alloy under quasi-static and dynamic conditions. Plastic flow of the ARMSTAL 30PM target material (parameters A, B, *n*) was defined according to the results of material characterization tests described in chapter 2.2.2. Strain rate hardening (parameter C), thermal sensitivity (parameter m), and the manner of failure of the material (value of failure strain ε_f_ = 0.78) were assumed to be similar to the other high-strength steels of 500 HB class and were adopted on the basis of the works [40,41,42,55,56,57], in which authors investigated the behavior of ARMSTAL 30PM and other armored steels with similar mechanical properties (Armox 500T, Ramor 500) using a combination of quasi-static and dynamic tests for a wide range of strain rates.

The A-IX-1 explosive composition filling the PG-7VM warhead was described with high-explosive burn (HEB) material model and Jones–Wilkins–Lee (JWL) equations of state (EOS) [38], which are often used to determine the pressure of the detonation products of the explosives in applications including acceleration of the metallic components [58,59,60]. The adopted parameters of the HEB material model and JWL EOS for the explosive used in simulations are shown in Table 11. The values were adopted on the basis of material characterization tests (described in Section 2.2.3) combined with Composition B (RDX is primer ingredient in Composition B) material model available in ANSYS AUTODYN material library [48] and information available in the literature [44,61,62,63]. In the work [61], authors used EXPLO-5 thermomechanical computer code [64] to predict detonation performance of A-IX-1 explosive composition. The calculation of detonation parameters was based on the chemical equilibrium steady state model of detonation by applying a modified White, Johnson, and Dantzig’s free energy minimization technique [65]. Experimental methods (X-ray investigation of the detonation wave in cylinders filled with water) were used in the work [44] to determine the detonation parameters of the phlegmatized RDX (composition similar to analyzed A-IX-1). Other thermochemical computer codes ZMWNI and CHEETAH were used by the authors in the work [63] for calculation of detonation parameters of RDX based composition.

The computational domain filled with air was described with a null material model combined with linear polynomial EOS (Table 12). The material allows EOS to be considered without computing deviatoric stresses. The linear polynomial EOS is linear in internal energy.

## 4. Results and Discussion

### 4.1. Results of Experimental Depth of Penetration Tests

**Figure 10:**
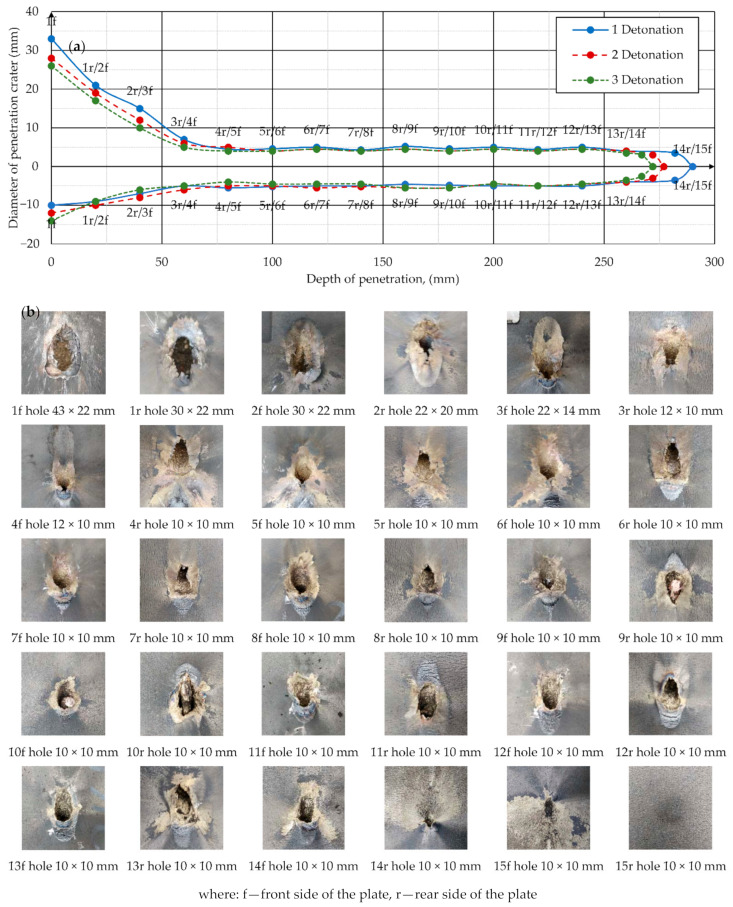
Results of DoP tests: (**a**) shapes of longitudinal cross-section of the penetration craters in the stack of ARMSTAL 30PM armored steel plates calculated on the basis of three experimental trials; (**b**) shapes of the inlet and outlet holes in the armor plates observed after the PG-7VM warhead detonation (2 trials).

The results of the DoP experimental tests are shown in Figure 10. Shapes of the penetration craters in the stack of ARMSTAL 30PM armored steel plates are shown in Figure 10a. The holes in the first three plates had an asymmetrical shape (Figure 10) due to the inclination of the warhead axis in relation to the vector normal to the surface of the target stack. The maximal dimensions of the inlet holes were 43 × 22 mm. The holes in the other plates had a nearly circular shape with the average diameter of 10 mm. Examples of shapes of the inlet and outlet holes in the individual plates obtained during the second detonation) are shown in Figure 10b. The stopped SCJ slug caught between the 9th and 10th plate can be observed in this figure. The average DoP calculated on the basis of three experimental trials (290 mm, 277 mm, 272 mm) was 280 mm. The results of the experiments were highly repetitive, which suggests that there were no factors (structural or material discrepancies) significantly affecting jet formation and penetration processes. Taking into consideration significantly higher mechanical properties of the ARMSTAL 30PM steel plates in comparison to the conventional RHA steel plates (ARMSTAL 30PM hardness was nearly double that of RHA), it can be concluded that the PG-7VM DoP capability in RHA is higher than the value of 300 mm that is the most frequently cited in the literature [30,31].

Determined average penetration capability of the PG-7VM warhead (DP = 280 mm) constituted the reference value in subsequent numerical reproduction of the SCJ formation and penetration analyses.

### 4.2. Results of Numerical Simulations

#### 4.2.1. Shaped Charge Jet Formation Process

**Figure 11:**
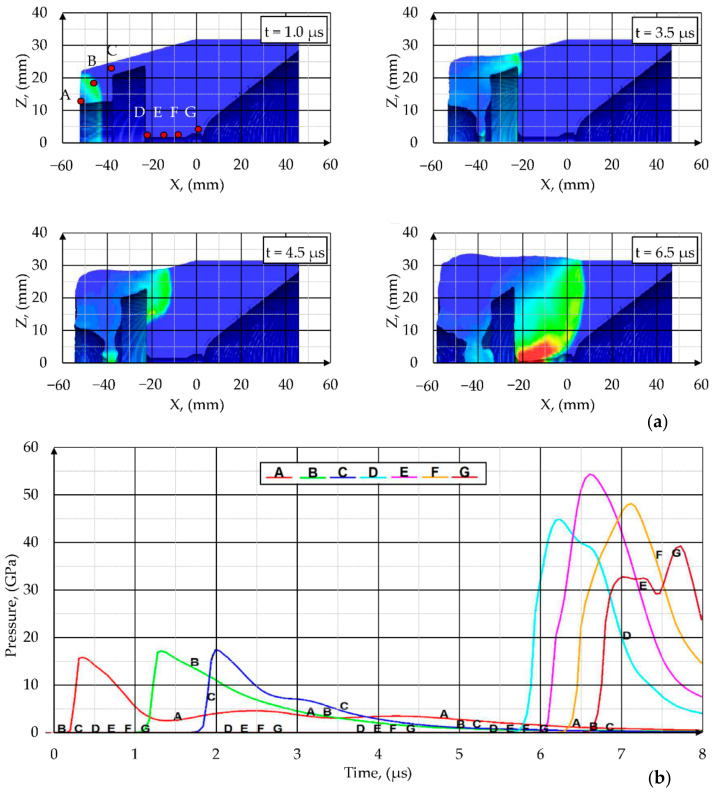
Propagation of the detonation wave in the PG-7VM warhead (**a**) and the pressure registered on the tracer nodes A–G (**b**).

The results of the simulation of SCJ formation due to the detonation of explosives in the PG-7VM warhead are presented in this chapter. The propagation of the shock front as well as expansion of the detonation products are shown in Figure 11 in X–Z coordinates. The pressure in the explosive was monitored at defined massless tracer nodes (points A-G in Figure 11a). After detonation, a spherical wave propagated outward from the point of initiation. The detonation wave moved through the explosive from the detonation point toward the liner surface at a very high velocity (close to the detonation velocity of the explosive used, approximately 6–8 km/s) and with pressure of 15–55 GPa. Time required for pressure wave to reach the liner surface was about 6 µs.

The influence of the wave shaper on the detonation wave propagation can be clearly seen in the Figure 11. The maximum pressure registered on tracer points increased nearly linearly with the distance from the detonation point up to the moment when the detonation wave reached the frontal surface of the wave shaper. The sudden boost of the pressure was observed at the tracer point D. The pressures registered at the tracers D–G were more than twice of those registered on tracers A–C. At the same time, tracer D was the first one in which pressure exceeded the Chapman–Jouguet pressure.

**Figure 12:**
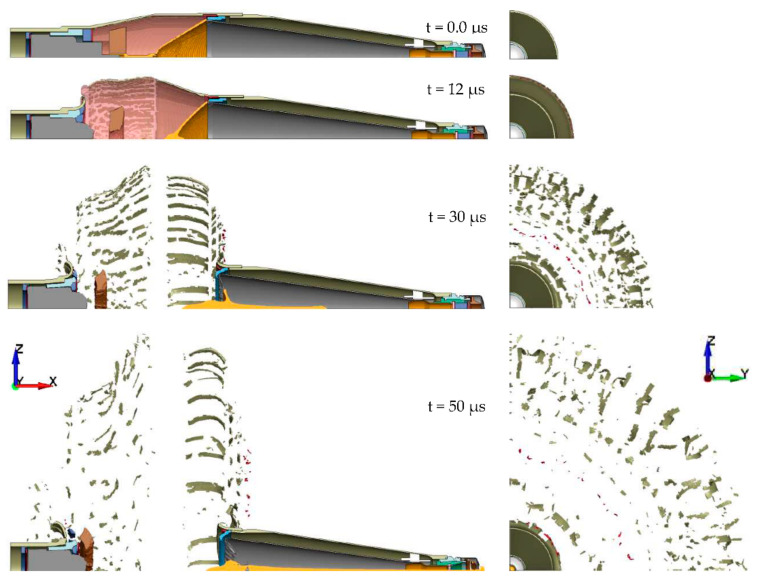
Deformation and damage of the PG-7VM warhead components during the SCJ formation process.

When the detonation front reached the liner, the liner was subjected to the intense pressure of the detonation front and began to collapse (Figure 12). During the process, the liner material was subjected to very large dynamic deformations with the strain rates of 10^4^–10^7^/s. The liner strains may exceed 1000% under very large hydrodynamic pressures (average pressure of approximately 15–55 GPa). The collapse of the liner material on the axis of the warhead forced a portion of the liner to form a high-velocity (2–8 km/s) jet. Since there was a large velocity gradient between the front (tip) and the rear part (slug) of the jet, it was stretched until it lost its integrity. At the same time, the expansion of the detonation products in the radial direction was evolving (Figure 12). That led to the expansion of the warhead body up to the moment of their fragmentation. Thus created fragments were moving in the radial direction at very high speeds exceeding 2 km/s. The fragments of the detonated warhead body may be considered as additional small penetrators and constitute a serious threat to the nearby personnel. At time t = 50 µs after the detonation of the warhead, the SCJ perforated the fuse cover in the grenade and the penetration of the target began.

**Figure 13:**
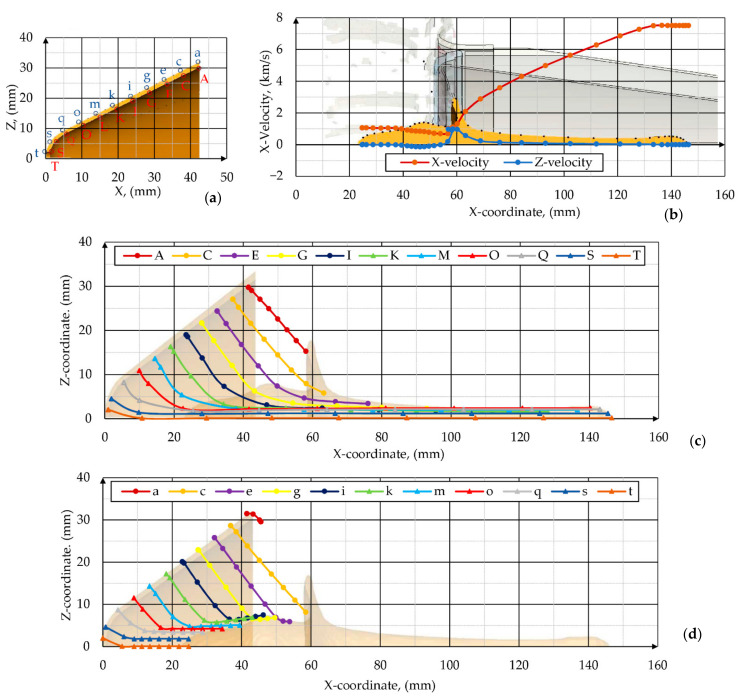
Analysis of jet formation process at time t = 30 µs after detonation: (**a**) initial locations of the tracer nodes; (**b**) jet velocity distribution; (**c**) trajectories of tracer points initially located on the inner surface of the liner; (**d**) trajectories of tracer points initially located on the outer surface of the liner.

Parameters of the SCJ that was formed from the detonation of the PG-7VM warhead were determined on the basis of the selected points on the inner (on the target side) and outer (on the explosive side) surface of the liner (points A-t and a-t in Figure 13a). Twenty-two massless tracer nodes (11 on the outer and 11 on the inner surface of the liner) were defined and their movement was tracked.

The locations of the points were updated according to the motion of the liner material. The histories of the points, including positions, velocities, stresses, plastic strain, volume fraction, and density, were recorded during the process. The gathered data were used in the analysis of the SCJ formation process. The shape of the SCJ of the PG-7VM warhead as well as its velocity distribution in axial and radial directions at randomly chosen time t = 30 μs after the detonation are shown in the Figure 13b. In accordance with PER theory [2], the jet velocity decreased monotonically from tip to tail. In Figure 13c the trajectories of the tracer points are plotted against time. On the basis of Figure 13, it is clearly visible that the high pressure of detonation products accelerated successive tracers on the liner. In consequence, the liner contour was changing. Each liner element was accelerated towards the axis of the PG7-VM warhead.

**Figure 14:**
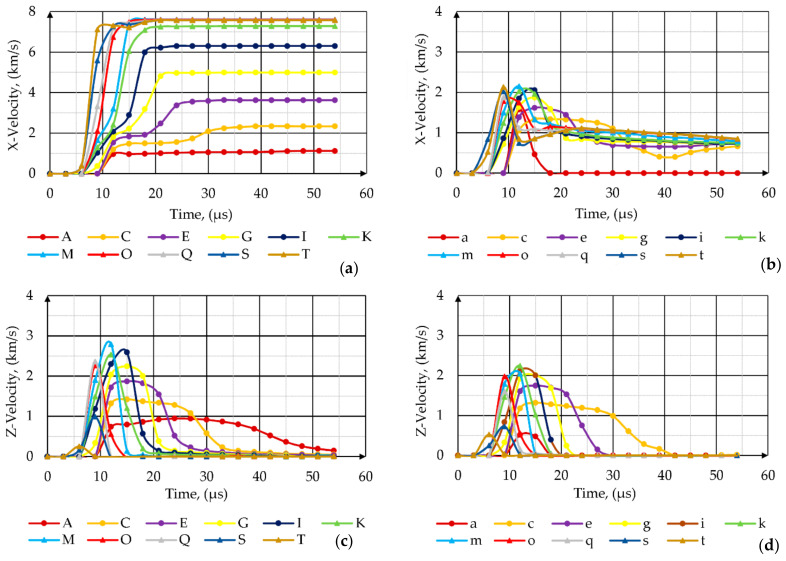
Axial velocities of tracer points initially located on the (**a**) inner and (**b**) outer surface of the liner. Radial velocities of tracer points initially located on the (**c**) inner and (**d**) outer surface of the liner.

The liner collapsed progressively from the apex to the base. On the basis of Figure 13 and Figure 14, it is clearly visible that all tracer points from the outer surface of the liner formed a slug. The axial velocity of those tracers did not exceed 2 km/s.

Taking into consideration information available in the literature that only the liner elements with the axial velocity greater than 2 km/s can effectively penetrate the armored steel target [1,67], it can be concluded that the velocities of the liner elements located between the A–C tracer nodes on the inner surface of the liner (Figure 14a) also had too low energy to contribute to the penetration of the target. Therefore, it seems that only about 30–40% of the liner volume went into the effective jet. The jet diameter was about one-tenth of the diameter of the cone. The jet tip velocity was slightly smaller than the detonation velocity of the explosive. The distribution of the jet velocity along its length was a nearly linear function from a maximum of 7.5 km/s at the tip down to about 2 km/s at the tail (rear) of the jet.

When analyzing the shapes of the axial velocity plots against time, the sudden boosts of acceleration of the liner elements right after they reached the area close to the warhead axis were visible, which testified to the concentration of energy in that zone. The axial velocity of the liner elements decreased from the apex (about 7 km/s and 2 km/s for the tracer nodes located on the inner and outer surfaces of the liner, respectively) to the base of the cone (about 2 km/s and 0 km/s for the tracer nodes located on the inner and outer surfaces of the liner respectively) (Figure 14a).

The radial velocities of the tracer nodes located on the inner and outer surface of the liner were plotted against time in Figure 14b. Tracer nodes from both sides of the liner were instantaneously accelerated to the axis. Their velocity increased linearly over a short period until it reached its maximum. Then, the velocity decreased as the nodes collapsed on the axis. Obviously, the tracer points from the inner side of the liner obtained higher radial velocities. The maximal obtained radial velocities of the points decreased together with the increase of the distance from the apex to the base of the liner direction.

#### 4.2.2. Shaped Charge Jet Penetration into Steel Target

**Figure 15:**
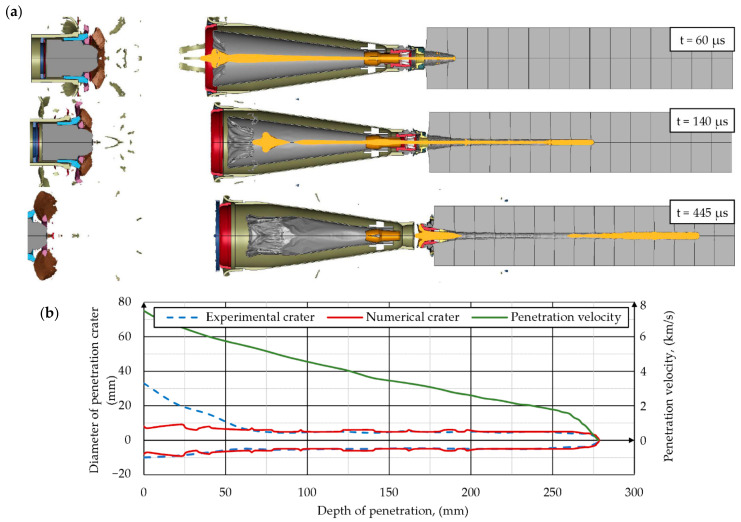
Results of simulations: (**a**) deformations of components at different times after the detonation of the PG-7M warhead; (**b**) penetration crater profiles.

**Figure 16:**
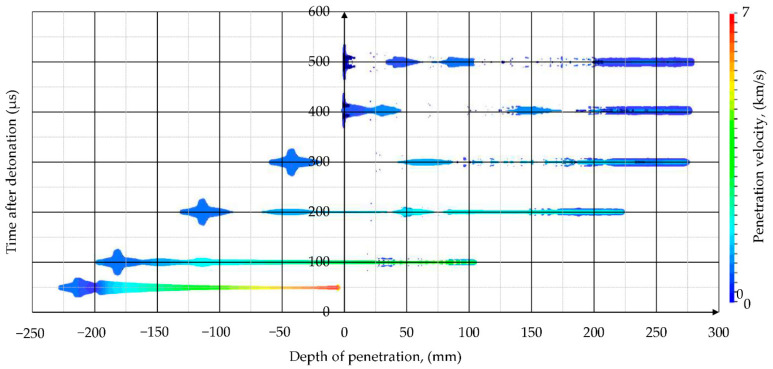
SCJ shapes and their velocity distribution at different times after the detonation initiation of the PG-7VM warhead.

**Figure 17:**
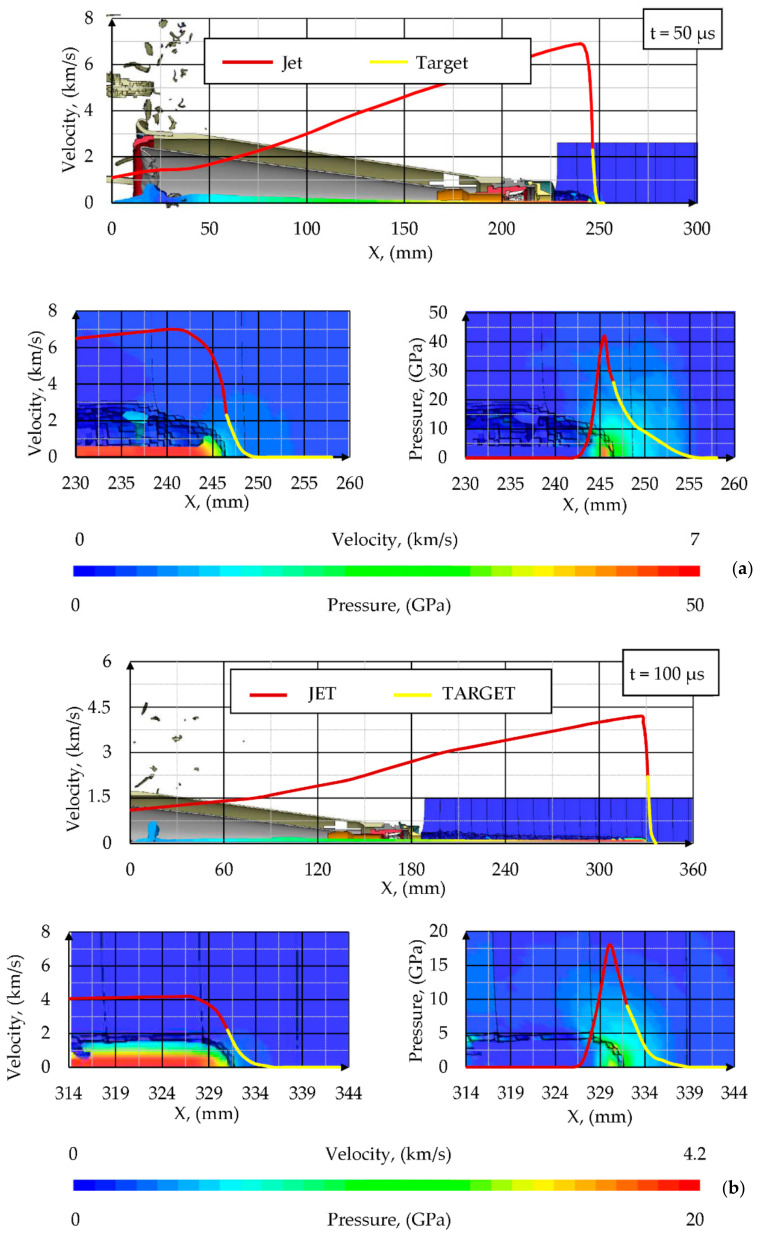
Distribution of jet velocity and pressure along the penetration axis at times (**a**) *t* = 50 µs and (**b**) *t* = 100 µs after detonation initiation of the PG-7VM warhead.

The results of simulation of SCJ penetration into a steel target are presented in this chapter. The deformations of the components of the simulation at different times after the detonation of the PG-7M warhead are shown in Figure 15a. The shapes of the penetration crater in the stack of ARMSTAL 30PM armored steel plates at different times are shown in Figure 15b. The jet formation analysis showed that the jet did not breakup before reaching the target. The SCJ shapes and their velocity distributions at different times after the detonation initiation of the PG-7VM warhead are shown in Figure 16. The hypervelocity of the SCJ generates pressures during jet-target impact that far exceed the yield strength of the target material. Peak pressures in the metal plate of 100–200 GPa were generated, decaying to an average of 10–20 GPa (Figure 17). The penetration process occurred at strain rates of 10^6^–10^7^/s. Therefore, the importance of target strength was significantly reduced in comparison to the impacts at lower velocities (*v* ≤ 1000 m/s). According to the information available in the literature, the SCJ penetration in armor plate is 10–15% [36] or even 20% [37] less than in mild steel.

Since two symmetry planes were used in the simulations and the warhead did not have any discrepancies, the SCJ traveled in a straight line. All the off-axis velocities of the jet particles were caused by the target reaction forces as well as the pressure of subsequent liner elements (Figure 17).

The cavity produced in the target was due to the lateral displacement of armor and jet elements by the high pressures that were generated. The penetration depth, hole dimensions and penetration velocities were measured in the simulations (Figure 15 and Figure 17).

Since in the simulations, two symmetry planes were used, the SCJ impact direction was perpendicular to the surface of the target and the holes in the first three plates did not have the asymmetrical shape like in experiments. The maximal dimensions of the inlet holes in the simulations were 16 × 16 mm. The holes in the other plates were circular with the diameter of approximately 10 mm. The depth of penetration in the simulation was DP = 278 mm. The difference between the experimental and numerical values was 2 mm (≤ 1%). 

The rear portion of the jet with the velocity lower than 2 km/s did not significantly contribute to the penetration. The final DoP = 278 mm was obtained at time t = 420 µs.

Axial velocity and pressure distribution in the SCJ and the target at selected moments of time are presented in Figure 17. Maximum velocity of the target was equal to minimal velocity of the penetrating SCJ (2.2 km/s) and constituted the “jet cut-off velocity”. SCJ elements with lower velocities than the cut-off velocity did not contribute to the target penetration. The SCJ was monotonically decelerated from the velocity of 7 km/s (Figure 17a) or 4.3 km/s (Figure 17b) to the penetration velocity of 2.3 km/s at the distance almost equal to the diameter of the SCJ.

**Figure 18:**
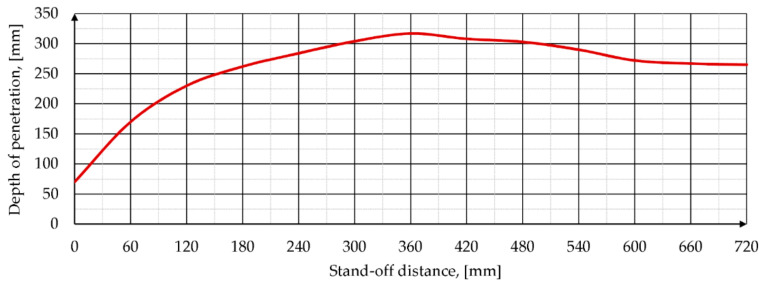
Characteristic of the PG-7VM warhead efficiency—DoP as a function of SoD.

A verified numerical model of the phenomenon of PG7-VM SCJ penetration into an ARMSTAL 30PM steel target was used to determine the basic characteristic of the PG-7VM warhead efficiency—DoP as a function of SoD. The relationship is shown in Figure 18. Together with the growth of the SoD, the number of elements in the numerical model and, in consequence, time required to finish the computations (CPU time) rapidly increased. For this reason, the 3D model with two symmetry planes had to be reduced to an axisymmetric formulation to keep the CPU time at reasonable level.

One of the consequences of using axisymmetric formulation is that some phenomena, e.g., lateral SCJ dispersion, could not be properly reproduced and the subsequent SCJ elements hit at the same point of the target as the previous elements. As a result, the plot of DoP as a function of SoD was similar to that of the perfect SC. Only the SCJ breakup mechanisms negatively affected the DoP of the target obtained at large SoD. However, the general tendency was determined and the maximum DoP = 317 mm was obtained for the SoD = 360 mm.

#### 4.2.3. Model Sensitivity Analysis

On the basis of the simulation results, it can be concluded that since the penetration crater in the ARMSTAL 30PM target was very deep (about 280 mm) and the SCJ interacted with a great number of target elements, the whole numerical model of the phenomenon may be very sensitive to the values of adopted target material failure criteria, as well as the methods of target discretization (mesh density). In the analyses, the value of effective plastic strain at failure (PSFAIL) was adopted as a target failure criterion. When the effective plastic strain in the element exceeded the defined limiting value of the PSFAIL parameter, the element was removed from the model. A set of additional simulations were carried out in which four different values of PSFAIL in the target material model were adopted (PSFAIL = 1.0; 0.8; 0.6; 0.4). The average value of failure strain was determined during the experimental compression tests and equaled ε_f_ = 0.65, while in the work [42] the authors reported failure strain of ARMSTAL 30PM of ε_f_ = 0.78.

**Figure 19:**
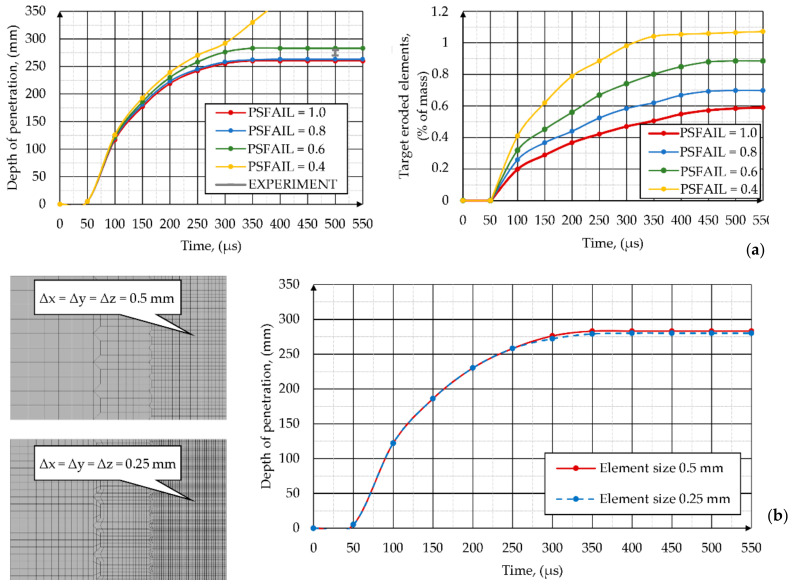
Influence of the value of effective plastic strain at failure in the target material model on (**a**) depth of penetration of the target and (**b**) target erosion ratio expressed in percent of mass; and influence of target mesh size on (**c**) depth of penetration of the SCJ.

The comparison of the simulation results in which different values of effective plastic strain at failure were adopted in the target material model are shown in Figure 19a.

On the basis of the performed calculations, it can be concluded that the model shows a relatively low sensitivity to changes of the PSFAIL value in the range of 0.1–0.6. Increasing the PSFAIL for the target material (ARMSTAL 30PM) by 33% from the value of PSFAIL = 0.6 to PSFAIL = 0.8 reduces the crater depth in steel plates by about 7% (from 280 mm to 260 mm). For higher PSFAIL values, the differences are even lower. The model shows higher sensitiveness when the PSFAIL value is reduced from 0.6 to 0.4. The target elements then erode much faster during the jet penetration process, which results in a complete penetration of the target with a thickness of 300 mm. The target penetration depth equal to DoP = 2 80 mm, i.e., the same as in the experimental tests, was obtained for the value of PSFAIL = 0.63. This value is slightly lower than that determined during the experimental compression tests of ARMSTAL 30PM steel samples (ε_f_ = 0.65) or available in the literature (ε_f_ = 0.78) [42].

Based on the results of analyses in which different sizes of finite element meshes were used in the target (Figure 19b), it should be stated that the model shows very low mesh sensitivity. The analyses were carried out for two sizes of the mesh of elements located in the area of direct impact of the SCJ: Δx = Δy = Δz = 0.5 mm and Δx = Δy = Δz = 0.25 mm. Therefore, the number of elements used in the target models were 343,200 and 2,745,600, respectively.

Taking into account the slight differences in the obtained calculation results (DoP = 283 mm and DoP = 278 mm for the elements of the size of 0.5 mm and 0.25 mm, respectively) and a large growth of required computational cost (time required to finish the computation increased from 17 to 38 h), use of elements with the size of Δx = Δy = Δz = 0.25 mm in analysis of this specific phenomenon seems to be unjustified from an economic point of view.

## 5. Summary and Conclusions

On the basis of literature review as well as performed experimental and numerical analyses the following conclusions can be drawn:

- In the literature, there is a lack of accurate and reliable research related to the performance of the most common PG-7VM warhead against modern high-hardness armored steels.
- In the present paper, the penetration capability of the PG-7VM warhead was determined against high-hardness (≥500 HB) ARMSTAL 30PM armored steel. The average depth of penetration of about 280 mm was obtained during the tests.
- In the present study, much attention was paid to exclude all the factors that could negatively affect (under- or overestimate) results of warhead penetration capability tests. Before the experiments, the presence of structural discrepancies in the warheads were X-ray inspected and material properties of explosive, target, and most important warhead components were analyzed during material characterization tests. The reliability of the results was significantly increased in this way.
- The numerical model of the warhead was defined more accurately than in previously published studies, since it was based on the real grenade dimensions and its technical documentation. Thanks to this, the difference between the experimental and numerical values of DoP was smaller than 1%, which confirms that developed methodology of modeling allows researchers to properly reproduce the PG-7VM shaped charge jet formation and target penetration processes.
- Performing numerical simulations facilitates a more precise analysis of the phenomenon and SCJ parameters. A verified numerical model of the PG-7VM SCJ penetration into ARMSTAL 30PM armored steel target was used to determine DoP in function of SoD. Maximum DoP = 317 mm was obtained for the SoD = 360 mm.
- A combined approach based on experimental tests and developed methodology of modeling of SCJ penetration into high-hardness armored steel targets could be used in the future in all kinds of optimization studies of different HEAT warheads and armor designs.

## Figures and Tables

**Table 1:** Chemical composition (%) of the analyzed ARMSTAL 30PM steel [35].

Steel Grade	C	Mn	Si	P	S	Ni	Cr	Mo	B	Cu	V	Ti
ARMSTAL 30PM	0.3	1.2	0.5	0.015	0.01	1.1	0.9	0.25	0.003	0.2	0.1	0.05

**Table 2:** Mechanical properties of ARMSTAL 30PM steel in relation to other steels with similar hardness (adapted from [35,39]).

Steel Grade	Thickness, (mm)	R_e02_, (MPa)	R_m_, (MPa)	A_5_, (%)	Impact Energy ISO-V-40 °C, (J)	Hardness, (HB)
ARMSTAL 30PM	Up to 20	1300	1600	**8**	>20	460 ÷ 540
Armox 600T		1500	2000	7	12	570 ÷ 640
Armox 500T	6 ÷ 13	1300	1500 ÷ 1750	8	20	480 ÷ 540
Armox 440T	up to 50	1100	1250 ÷ 1550	10	30	420 ÷ 480
Hardox 400	up to 50	1000	1250	10	30	360 ÷ 440
Hardox 500	up to 50	1300	1550	8	25	450 ÷ 530
Weldox 900E	up to 25	960	1060	14	30	-
HCM 480 MILAR	8 ÷ 40	1250	1450 ÷ 1750	8	min. 20	480 ÷ 520
HCM 580 MILAR	8 ÷ 40	1500	2000	7	min. 12	580 ÷ 620

**Table 3:** The results of ARMSTAL 30PM hardness measurements and impact strength tests.

No.	Steel Grade	Hardness (HBW)		KCV_−40 °C_ (J/cm^2^)	KCU_+20 °C_ (J/cm^2^)
		Parallel to Rolling Direction	Perpendicular to Rolling Direction		
1	ARMSTAL 30PM–4 mm	506	498	78	140.3
2	ARMSTAL 30PM–6 mm	512	502	111	183
3	ARMSTAL 30PM–8 mm	508	502	110	184

**Table 4:** Dimensions of the ARMSTAL 30PM samples prepared for the mechanical tests.

**Steel Grade**	**Dimensions (mm)**							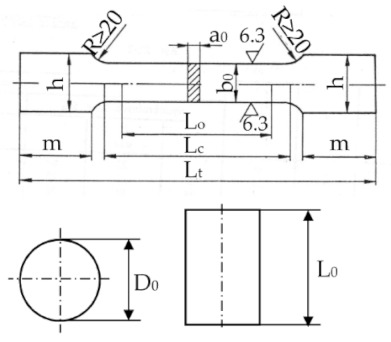
	**a_0_**	**b_0_**	**h**	**m_min._**	**L_0_**	**L_c min._**	**L_t min._**	
**ARMSTAL 30PM**	4.0	20	30	60	50	65	215	
	6.0	20	35	70	70	90	260	
	8.0	15	35	70	80	100	270	
	**D_0_**	**L_0_**						
	4.0	8.0						
	6.0	10.0						
	8.0	10.0						

**Table 5:** Values of mechanical parameters of the ARMSTAL 30PM determined during the tests.

No.	Steel Grade	Density (kg/m^3^)	Young Modulus (GPa)	Poisson Ration (ν)	Tension			Compression		
					R_m_ (MPa)	R_e_ (MPa)	A_5_ (%)	R_m_ (MPa)	R_e_ (MPa)	ε_f_ (%)
1	ARMSTAL 30PM–4 mm	7800	210	0.32	1642	1401	10.2	1780	1523	0.71
2	ARMSTAL 30PM–6 mm	7800	210	0.32	1623	1385	12.8	1765	1510	0.63
3	ARMSTAL 30PM–8 mm	7800	210	0.33	1645	1363	10.15	1782	1501	0.61

**Table 6:** Values of parameters obtained during DSC and TGA analyses for samples of A-IX-1 material extracted from PG-7VM warheads.

Batch No.	Measurement	Melting			Decomposition (DSC)			Decomposition (TGA)		
		T_onset_ (°C)	T_max_ (°C)	T_onset_ (°C)	T_end_ (°C)	Δm (%)	Q (J/g)	T_onset_ (°C)	T_max_ (°C)	Q (J/g)
X–90–406	1	204.2	205.0	221.0	248.6	98.7	−178.1	217.1	242.2	1986
	2	204.1	205.1	220.8	248.8	99.0	−177.3	217.3	243.0	2012
Y–85–406	1	204.0	205.2	219.0	248.0	97.5	−174.6	218.6	244.0	2363
	2	204.3	205.4	219.1	248.3	97.2	−175.8	219.5	244.6	2411
Z–78–406	1	204.2	204.9	217.2	248.3	95.9	−181.9	217.2	243.5	2507
	2	204.1	205.1	217.5	248.8	96.4	−181.1	217.8	243.9	2491

**Table 7:** Results of density, moisture, and stability analyses of A-IX-1 explosive composition samples extracted from PG-7 warheads.

Batch No.	Measurement	Density (g/cm^3^)	Humidity (%)	Stability (%)
X-90-406	1	1.73	0.03	0.05
	2	1.72	0.03	0.05
Y-85-406	1	1.71	0.04	0.06
	2	1.70	0.04	0.07
Z-78-406	1	1.70	0.03	0.03
	2	1.70	0.04	0.04

**Table 8:** Detonation wave propagation velocity of A-IX-1 explosive composition in function of its density [42,43].

Density, ρ, (g/cm^3^)	1.25	1.30	1.35	1.40	1.45	1.50	1.55	1.60	1.63
Detonation velocity, D, (m/s)	6660	6875	7125	7315	7470	7640	7820	7.995	8300
	[43]								[44]

**Table 9:** Parameters of the MMALE and fluid–structure interaction keywords used in the simulations [45].

**Advection Logic**	**Advection Frequency**	**Advection Method**	**Bucket Sort Frequency**	**Timestep Safety Factor**	**Hourglass**
DCT = −1	NADV = 1	METH = −2	NBKT = 10	TSSFAC = 0.4	IHQ = 1
**Coupling Points**	**Coupling Method**	**Coupling Direction**	**Min. Volume Fraction**	**Stiffness Factor**	**Leakage Control**
NQUAD = 2	CTYPE = 5	DIREC = 1	FRCMIN = 0.1	PFAC = 0.1	ILEAK = 2

**Table 10:** Material parameters of the metallic components of the PG-7VM warhead used in simulations.

Component	Material	RO, (g/cm^3^)	G, (GPa)	PR	A, (MPa)	B, (MPa)	n	C	m	C_grun_, (m/s)	S1	γ	SOURCE
Liner, conductor	Copper	8.96	46	0.33	90	292	0.31	0.025	1.09	3940	1.49	2.02	[48]
Body, ballistic cap, etc.	Al alloy	2.785	27.6	0.31	352	440	0.42	0.015	1	5328	1.33	2.0	[48,53,54]
Target	ARMSTAL 30PM	7.85	77	0.33	1345	575	0.19	0.001	1	4610	1.73	1.67	ADOPTED
					1875	415	0.98	0.001	1				[40,41,42]
	Armox 500T	7.85	77	0.33	1470	702	0.199	0.0054	0.81				[55]
					1540	332	0.26	0.013	1.2				[56]
	Ramor 500	7.85	67	0.33	1021	965	0.057	0.001	0.84				[57]

where: RO—density; G—shear modulus; PR—Poisson’s ratio; A, B, *n*, C, m—parameters of JC material model; C_grun_, S1, γ —parameters of Gruneisen’s EOS [46].

**Table 11:** Material parameters of the metallic components of the PG-7VM warhead used in simulations.

	RO, (g/cm^3^)	D, (m/s)	PCJ, (GPa)	A, (GPa)	B, (GPa)	R1	R2	ω	EO, (kJ/m^3^)	VO	Source
A-IX-1	1.65	8240	26.9	880	12.7	5.0	1.1	0.39	8500	1	Adopted
COMP B	1.717	7980	29.5	524	7.67	4.2	1.1	0.34	8500		[48]
RDX_fl_	1.63	8225	26.3						5500		[44]
RDX_ZMWNI_		8267		989.1	11.12	5.16	1.04	0.396	9417		[63]
RDX_CHEETAH_		8266		828.1	10.527	4.84	1.06	0.395	9357		[63]
A-IX-1	1.66	8258	27.35	887.8	23.826	5.18	1.6	0.45	9190		[61]

where: RO—density; D—detonation velocity; PCJ—Chapman–Jouguet pressure; A, B, R1, R2, ω, E0, V0—parameters of JWL EOS [46].

**Table 12:** Material parameters of the air filling computational domain used in simulations.

RO, (g/cm^3^)	C0	C1	C2	C3	C4	C5	C6	E0	Source
1.29 × 10^−3^	0	0	0	0	0.4	0.4	0	0	Adapted from [66]

where: C1, C2, C3, C4, C5, C6—polynomial equation coefficients in linear polynomial EOS; E0—initial internal energy per unit [46].

## Data Availability

Data sharing is not applicable to this article.
